# A Rare Case Report of Trichilemmal Carcinoma

**DOI:** 10.7759/cureus.31261

**Published:** 2022-11-08

**Authors:** Chafik Rhoul, Achraf Miry, Amal Bennani, Badr Serji, Tijani El Harroudi

**Affiliations:** 1 Surgical Oncology, Mohammed First University, Faculty of Medicine and Pharmacy, Oujda, MAR; 2 Pathology, Mohammed First University, Faculty of Medicine and Pharmacy, Oujda, MAR; 3 Anatomopathology, Mohammed First University, Faculty of Medicine and Pharmacy, Oujda, MAR

**Keywords:** sebaceous gland, sun-exposed area, surgical excision, nodule-ulcerated lesion, skin adnexal tumor

## Abstract

Trichilemmal carcinoma (TC) is a rare skin malignant tumor with pillar differentiation. TC presents along with other malignant hair follicle tumors and accounts for only 1% of all adnexal carcinomas. TC usually occurs on sun-exposed skin in elderly people, nevertheless, it can occur at any age. We report a case of trichilemmal cyst carcinoma in a 54-year-old woman presenting with an increasing occipital cyst. A histological examination confirmed the diagnosis and a large excision was performed. Despite the absence of a well-defined consensus on the management of TC, surgical excision with adequate margins seems to be safe in the absence of metastatic lesions. However, in the case of second localization, chemotherapy could be initiated, but again, in this case, no consensus on the appropriate protocols exists.

## Introduction

Trichilemmal carcinoma (TC) is a rare tumor that usually occurs on sun-exposed skin, especially on the face, scalp, neck, and back of the hands. They are usually seen in elderly subjects (commonly between the 4th and 9th decades of life) but can develop at any age [1]. The exact pathogenesis of TC is not yet known, but most patients have a history of significant lifetime sun exposure [2]. The first case reported in the literature was described by Headington in 1976 [3]. TC originally develops from either sweat glands, follicles, and/or sebaceous glands [4]. We report a case of this rare skin tumor that is not yet widely known

## Case presentation

**Figure 1:**
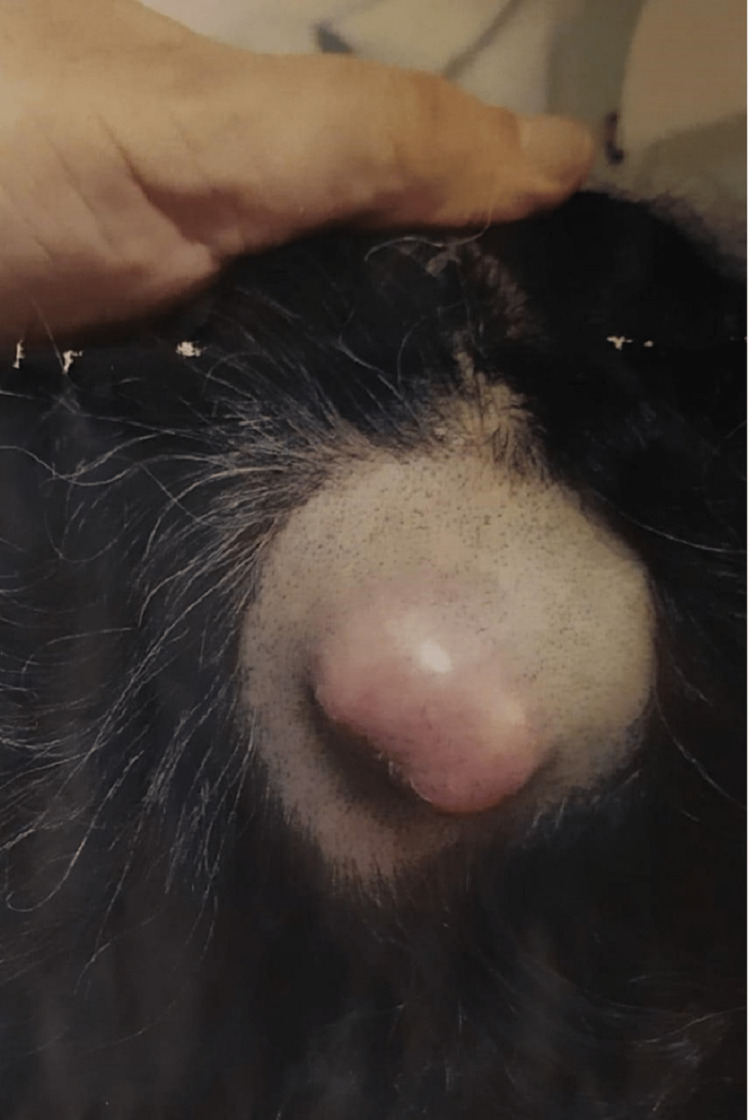
Preoperative photograph before the first surgery

A 54-year-old White North African woman, with no medical history, reported a nodule on her scalp which was discovered by herself six months ago. A partial cyst exeresis was performed with a pathological diagnosis of TC with positive lateral and deep surgical sections. The patient was then referred to our oncological and general surgery department for further care. The clinical examination noted a tumefaction of the scalp located in the occipital region with the absence of a second location (Figure 1). Cerebral CT scan findings showed a left occipital thickening measuring 10*14mm with no other localization.

**Figure 2:**
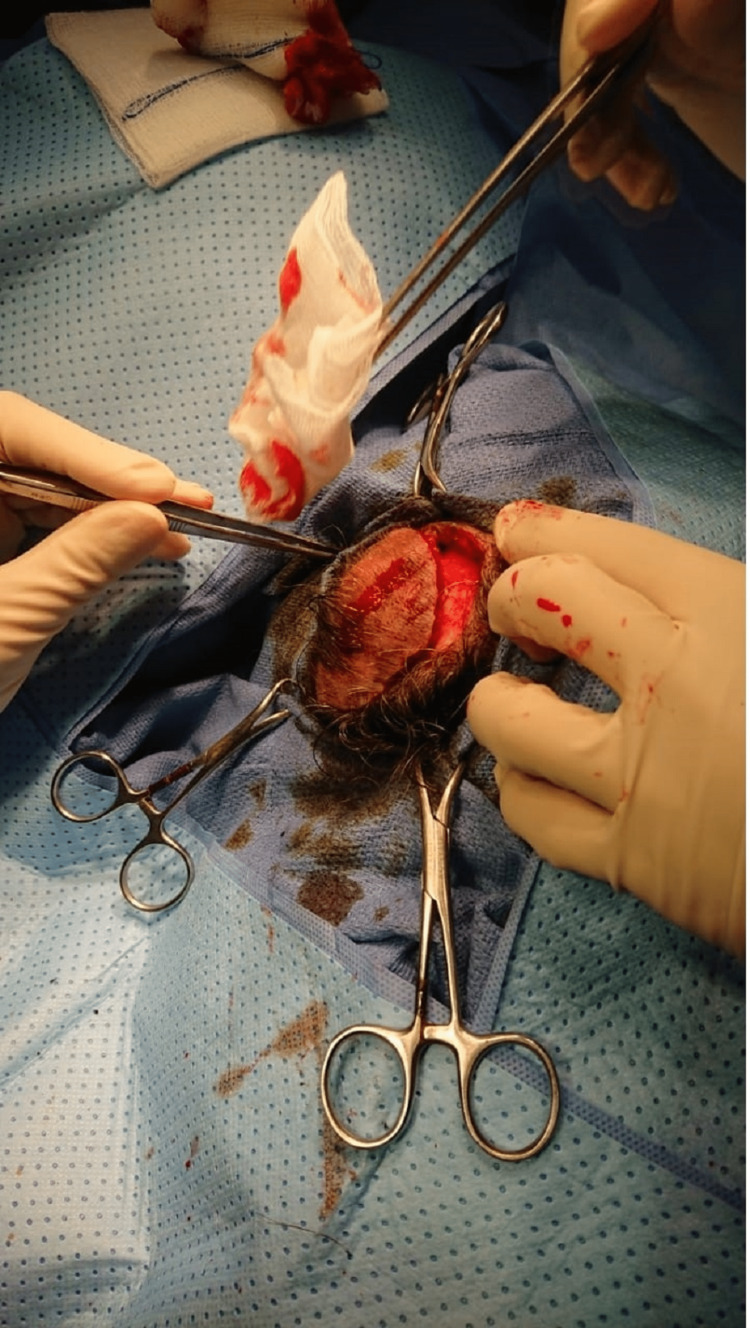
Large resection of the tumor

**Figure 3:**
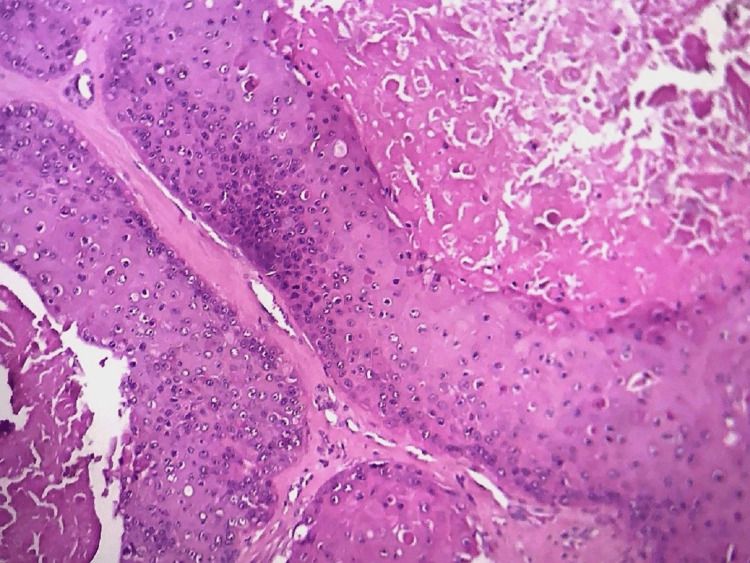
Microphotography showing a proliferation with two different aspects; a carcinomatous proliferation made of glands (right field) and a squamous proliferation with little cytonuclear atypia (left field); H&E 200x

**Figure 4:**
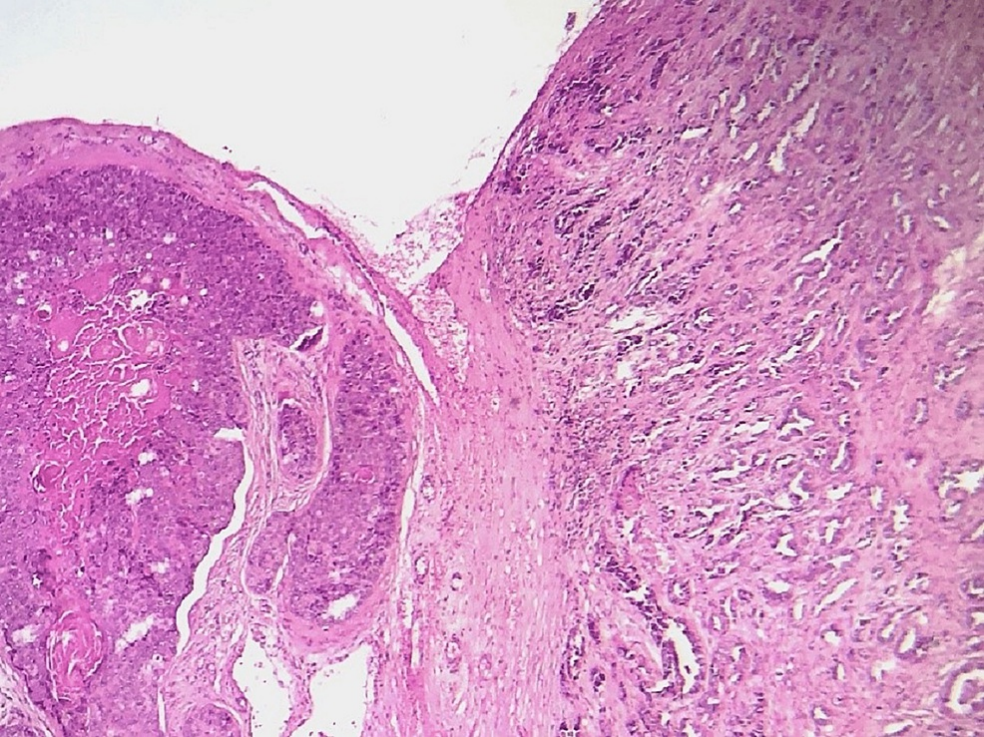
Microphotography showing a carcinomatous proliferation made of irregular tubes; the neoplastic cells are markedly atypical (H&E 400x)

**Figure 5:**
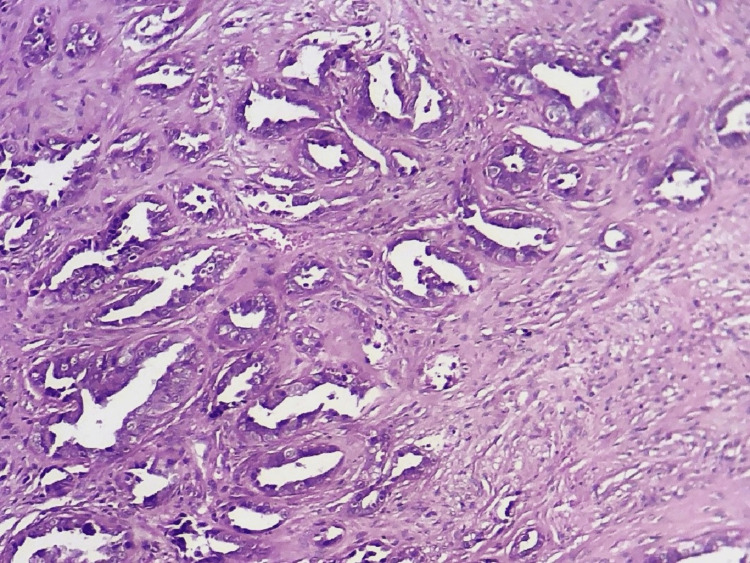
Microphotography showing a squamous cystic wall made of cells showing mild atypia and production of eosinophilic dense keratin (H&E 400x)

A large tumor exeresis was performed two months after the first excision (Figure 2). The histopathological diagnosis showed a tumoral proliferation with no vascular embolism and clean surgical sections (Figures 3-5). One month after surgery, the patient was seen again and clinical examination showed a good cicatrization process with no physical complaints. Six months later, the scar healed and a cervical and thoracic CT scan was performed; no suspicious lesions were found.

## Discussion

TC is a rare tumor that usually occurs in sun-exposed areas in elderly persons with no apparent predilection for either sex [1]. Though the pathophysiology of TC remains poorly defined, there are certain factors attributed to TC such as ultraviolet ra­diation, immunosuppression, skin traumatisms (burns, scars), and genetic diseases like xeroderma pigmentosum and Cowden syndrome [4]. The duration from tumor growth to clinical manifestation can range from two months to 50 years; faster tumor growth has been noted in some patients. Clinically, these tumors were often considered benign lesions [2]. The lesions are frequently solitary presentations and may present as nodules, plaques, or ulcerated lesions [5]. Its differential diagnosis is made with other skin tumors like squamous cell carcinoma, basal cell carcinoma, keratoacanthoma, and malignant nodular melanoma [6-7].

Histologically, TC develops from the external epithelial sheath of the hair root with a well-limited and infiltrating lobule proliferation comprising large cells with a clear cytoplasm related to the accumulation of intra-cytoplasmic glycogen. Areas of hemorrhage and/or necrosis can be seen in large size tumors. Therefore, the mitotic activity of the cells is intense with several signs of atypia, and they also colorize very well on periodic acid-Schiff (PAS) stains [5]. On immunohistochemistry, TC is positive for cytokeratin CK1, 10, 14, 17, and 19 and is negative for the carcinoembryonic antigen (CEA), although late positive results have occasionally been reported [6,8]. They are also negative for the S-100 antigen and the other cytokines (CK 7, 8, 15, 16, 18) [9].

The treatment is exclusively surgical, although no consensus on managing TC is available [10]. Simple excision with adequate margins seems to be safe [11,12]. Lymphatic nodes and distance metastasis are rare and require systemic chemotherapy, but no standardized protocol is currently available [10]. It turns out that there are some chemotherapy trials for metastatic forms described in the literature. Xu et al. [2] suggest systemic chemotherapy with four cycles of cisplatin and vindesine or four cycles of cisplatin and cyclophosphamide as it may control the progression of the disease [4].

TC generally has a good prognosis; reports of local recurrence cases are uncommon [13,14]. The critical prognostic factors affecting survival are safe surgical margin and lymph node metastasis [10]. TC has a slow evolution and after complete excision, there is rarely a local recurrence or distant metastases [2]. The patient should be followed up closely to identify any future recurrences.

## Conclusions

Despite the absence of a well-defined consensus on the management of TC, surgical excision with adequate margins seems to be safe and effective in the absence of metastatic lesions. In the case of second localization, chemotherapy could be initiated, but again, in this case, no consensus on the appropriate protocols exists.
